# Sleep disturbance and multimorbidity: a cross-sectional and longitudinal study in the knee pain and related health in the community cohort

**DOI:** 10.1093/sleepadvances/zpaf039

**Published:** 2025-06-11

**Authors:** Will Thompson, Subhashisa Swain, Carol Coupland, Frances Rees, Phil Courtney, Michelle Hall, Eamonn Ferguson, David A Walsh, Ana M Valdes, Richard Morriss, Michael Doherty, Weiya Zhang

**Affiliations:** Academic Rheumatology, Injury Recovery and Inflammation Sciences, School of Medicine, University of Nottingham, Nottingham, UK; Pain Centre Versus Arthritis, University of Nottingham, Nottingham, UK; Academic Rheumatology, Injury Recovery and Inflammation Sciences, School of Medicine, University of Nottingham, Nottingham, UK; Pain Centre Versus Arthritis, University of Nottingham, Nottingham, UK; School of Medicine, Keele University, Keele, UK; Centre for Academic Primary Care, School of Medicine, University of Nottingham, Nottingham, UK; Academic Rheumatology, Injury Recovery and Inflammation Sciences, School of Medicine, University of Nottingham, Nottingham, UK; Nottingham University Hospitals NHS Trust, Nottingham, UK; Nottingham University Hospitals NHS Trust, Nottingham, UK; School of Psychology, University of Nottingham, Nottingham, UK; School of Psychology, University of Nottingham, Nottingham, UK; Academic Rheumatology, Injury Recovery and Inflammation Sciences, School of Medicine, University of Nottingham, Nottingham, UK; Pain Centre Versus Arthritis, University of Nottingham, Nottingham, UK; NIHR Nottingham Biomedical Research Centre, Nottingham, UK; Academic Rheumatology, Injury Recovery and Inflammation Sciences, School of Medicine, University of Nottingham, Nottingham, UK; Pain Centre Versus Arthritis, University of Nottingham, Nottingham, UK; NIHR Nottingham Biomedical Research Centre, Nottingham, UK; Institute for Mental Health, University of Nottingham, Nottingham, UK; Academic Rheumatology, Injury Recovery and Inflammation Sciences, School of Medicine, University of Nottingham, Nottingham, UK; Pain Centre Versus Arthritis, University of Nottingham, Nottingham, UK; NIHR Nottingham Biomedical Research Centre, Nottingham, UK; Academic Rheumatology, Injury Recovery and Inflammation Sciences, School of Medicine, University of Nottingham, Nottingham, UK; Pain Centre Versus Arthritis, University of Nottingham, Nottingham, UK; NIHR Nottingham Biomedical Research Centre, Nottingham, UK

**Keywords:** KPIC, sleep disturbance, multimorbidity, longitudinal

## Abstract

**Study Objectives:**

To examine whether there is a temporal association between sleep disturbance and multimorbidity.

**Methods:**

We performed a cross-sectional and longitudinal observational analysis in people aged 40 years or more, recruited from the knee pain and related health in the community cohort study. The primary exposure was the Sleep Problems Index II score in tertiles measured at baseline. The primary outcome was count of chronic conditions developed in 5 years. Pain, low mood, and anxiety were measured at 2 years as mediators. Poisson regression was used to calculate adjusted relative risk and 95% confidence intervals.

**Results:**

We included 4488 participants in the cross-sectional analysis at baseline and 1941 in the 5-year longitudinal analysis. At baseline, the adjusted relative risks for prevalent multimorbidity were 1 (reference) for tertile 1, 1.09 (95% confidence interval; 1.01–1.18) for tertile 2, and 1.21 (95% confidence interval; 1.11–1.32) for tertile 3 of the sleep disturbance score (p for trend <.001). Of the total association between sleep disturbance and multimorbidity, 14 per cent (95% confidence interval; 9% to 19%) were mediated by pain and 7 per cent (95% confidence interval; 2% to 13%) by low mood. In the 5 year follow-up, the adjusted relative risk for incident multimorbidity were 1 (reference) for tertile 1, 1.12 (95% confidence interval; 0.98–1.28) for tertile 2, and 1.25 (95% confidence interval; 1.06–1.47) for tertile 3 (p for trend .007). Of the total association between sleep disturbance and multimorbidity, 10 per cent (95% confidence interval; 2% to 18%) was mediated by pain.

**Conclusions:**

Sleep disturbance is associated with multimorbidity. The association is dose-dependent, temporal, and partially mediated by pain.

**Statement of Significance**

Sleep disturbance has been hypothesized to be a risk factor for the development of multimorbidity. We investigated this association using data from participants aged 40 and over in the knee pain and related health in the community cohort. We found that sleep disturbance is indeed associated with both prevalent and incident multimorbidity. The association is dose-dependent and temporal, and partly mediated by pain.

## Introduction

The growing impacts of multiple long-term health conditions within the same individual, i.e. multimorbidity, are concerning for both patients and the healthcare providers. DuGoff et al. [1] found that people aged over 67 years old with 10 or more long-term health conditions live on average 18 years less than similar individuals without any long-term health conditions, and that life expectancy decreased as the number of conditions increased. A systematic review in 2021 found that individuals with 7–9 long-term conditions cost the healthcare system 3.82 (95% CI; 3.01–4.85) times more than individuals without multiple long-term conditions [2], and a Finnish study found that patients classed as “multimorbid at risk” accounted for 62 per cent of total healthcare costs [3]. Therefore, interventions to reduce the incidence of multimorbidity should reduce the number of consultations, hospitalizations, and healthcare costs.

Sleep disturbance can be defined as the total accumulation of sleep-specific traits that interfere with an individual’s ability to get restful, restorative sleep that allows them to remain active while awake. It has been associated with multimorbidity, but the temporal association with this outcome is unclear [4]. However, sleep disturbance is associated with chronic pain, depression, and anxiety [5], which in turn are associated with multimorbidity [6–9], and some of these associations are likely causal [10].

The relationship between sleep disturbance and pain is bidirectional, with pain disrupting sleep but good or poor sleep being associated with improvements or worsening of pain [11]. There is robust evidence that interventions to improve sleep have moderate to large effects on sleep symptoms with some functional benefits but relatively small benefits for the other symptoms of depression, anxiety, and other mental disorders [12–14]. However, there may be a subgroup of people with depression who derive deeper benefit for their depression with treatments for insomnia, though the clinical characteristics and underlying pathophysiology of this important subgroup are yet to be clearly defined [14]. In contrast, interventions for insomnia in people with pain improve not only sleep disturbance but also pain and depression, although the effects on anxiety are inconsistent [15, 16]. These studies suggest that sleep disturbance is a key factor in the network of long-term health conditions. However, whether sleep disturbance is directly associated with multimorbidity, or indirectly through pain, depression, or anxiety has not been fully investigated.

To address this gap, we undertook this study to investigate whether sleep disturbance is associated with current multimorbidity and whether it can predict incident multimorbidity prospectively. Given that sleep disturbance is associated with pain, depression, and anxiety, it is possible that these conditions could be confounding variables between sleep disturbance and multimorbidity. However, it is also possible that sleep disturbance is associated with these conditions, which in turn leads to multimorbidity. Therefore, we also investigated whether any of the associations we found between sleep disturbance and multimorbidity are mediated by multisite chronic pain, low mood, and anxiety.

## Materials and Methods

This study was approved by the Nottingham Research Ethics Committee 1 (NREC reference 14/EM/0015).

### Study design

**Figure 1:**
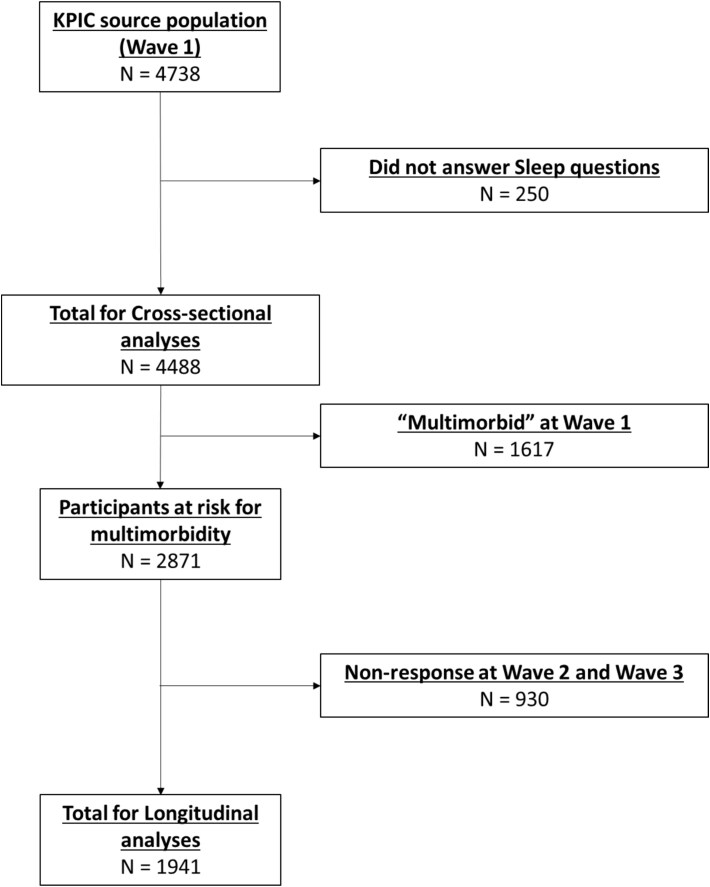
Selection of participants.

We carried out a cross-sectional and longitudinal study using data obtained from participants in the knee pain and related health in the community (KPIC) cohort. KPIC was a cohort study designed primarily to investigate prevalence and incidence of knee pain and its related health conditions among people aged 40 and over around the city of Nottingham. As a result, data for knee pain and its related health conditions have been collected, which provided an excellent opportunity to study multimorbidity. Between March 14 and December 1, 2014, 40 505 questionnaires were sent to all men and women (irrespective of having knee pain) over the age of 40 who were registered at 12 general practices in the East Midlands in England, of whom 9514 returned the questionnaire [17, 18]. The cohort was assessed at three waves (wave 1 [2014–2015], wave 2 [2017], and wave 3 [2020]). Long-term conditions at wave 1 were accumulated to calculate the multimorbidity score for the cross-sectional analysis at wave 1 and to identify participants at risk for incident multimorbidity during the follow-up for the longitudinal analysis (Fig. 1).

### Exposure

For our primary exposure of sleep disturbance, we used the Sleep Problems Index II score based on 9 out of 12 questions from the MOS (Medical Outcome Study) scale, with higher values meaning increasing levels of sleep disturbances [19]. Sleep Problems Index II mainly focusses on difficulty falling asleep, restless sleep, tiredness during the day, problems breathing during sleep, and self-reported dissatisfaction with sleep. The answers, initially scored from 1 to 5 or 1 to 6, were then transformed into a scale from 0 to 100, and the final value of the score is the average value (0–100) of all 9 questions [19]. To investigate the relationship between sleep disturbance and multimorbidity in a clinically meaningful manner, we divided the wave 1 participants into three groups based on the tertiles of the Sleep Problems Index II score: tertile 1 (T1, <20.6), tertile 2 (T2, >20.6 and <37.8), and tertile 3 (T3, >37.8), such that the higher the sleep tertile, the greater the level of sleep disturbance. This is a standard way of transforming a continuous exposure when measuring its association with a non-continuous outcome.

### Outcome

For our primary outcome, we used a multimorbidity count based on a list of 38 common health conditions previously used for KPIC research [18], specifically: joint pain (cause unspecified), spinal problems, liver, and gallbladder disorders, psoriasis, migraines, neurological disorders, psychological problems, vitamin D deficiency, coeliac disease, rheumatoid arthritis, psoriatic arthritis, systemic lupus erythematosus, lymphatic system disorders, peripheral vascular disease, polymyalgia, Parkinson’s disease, eye problems, epilepsy, sleep problems, gout, chronic obstructive pulmonary disorder, skin disorders, colitis, prostate disorders (not cancer), upper gastrointestinal disorders, kidney disorders, hearing problems, cancer (all types), hernias, osteoporosis, thyroid disorders, heart disease, hypertension, osteoarthritis, diabetes, stroke, irritable bowel syndrome, fibromyalgia, and chronic fatigue syndrome. The multimorbidity count was calculated by adding together the number of reported health conditions for each participant, which was done at waves 1–3.

### Covariates

#### Demographics

Age [20], sex [21], body mass index (BMI) [22], and neighborhood deprivation [23] are widely reported risk factors for chronic health outcomes; thus, we decided to include them as covariates in our analysis. Data were available for basic demographic information (e.g. age, sex, BMI) which was self-reported by participants. There were also data for the self-reported number of different medications taken by participants. Information about each participant’s neighborhood deprivation was measured using the Index of Multiple Deprivation rank (with 1 being the most deprived and 10 being the least deprived) [24] which was derived from an online database based on the participants post-code [25].

#### Hospital anxiety and depression scale

Depression [26, 27] and anxiety [27] are also widely reported predictors of chronic health outcomes, so we decided to investigate them, as covariates as well as mediators. Participants scores for low mood (a surrogate measure through questionnaire for depression) and anxiety were measured using the Hospital Anxiety and Depression scale [28], where participants complete a questionnaire about how they have felt in the past week, with their answers (relating to low mood and anxiety, respectively) added up into a scale ranging from 0 to 21.

#### Body pain map

Multisite chronic pain [29] is also widely reported to be a predictor of chronic health outcomes; therefore, we decided to investigate it as both a covariate and a mediator. Participants were also asked to report whether they had pain at any site of their body at any time during the previous 4 weeks. This was done using a body map, in which participants shaded the areas on the map which matched the parts of their body where they felt pain; the researchers independently categorized the body map into 45 different regions, allowing them to assign a value of one if that region was shaded in. The number of pain sites were added together to make a “multisite chronic pain score.”

### Statistical analysis

When summarizing the data, age and BMI were presented as mean and standard deviation (SD), sex, and medication count were presented as percentages; and the multimorbidity, low mood, anxiety, and multisite chronic pain scores, as well as the Index of Multiple Deprivation rank, were presented as median and interquartile range as they were not normally distributed. To compare differences between the tertiles of the sleep disturbance scale, the Analysis of Variance (ANOVA) test was used for age and BMI, the chi-squared test was used for sex and medication count and the Kruskal–Wallis test was used for the low mood, anxiety, and multisite chronic pain scores and Index of Multiple Deprivation rank.

For the cross-sectional analysis at wave 1, we investigated the association between sleep disturbance in tertiles and the multimorbidity score as a count variable (i.e. 0, 1, 2, 3, 4, …, etc.) according to the number of long-term conditions a participant had at wave 1. Multivariable Poisson regression was used to estimate the rate ratios (RRs) and 95% confidence intervals (95% CIs). We used the lowest tertile of the sleep disturbance score (i.e. the lowest level of sleep disturbance) as the reference group to calculate the RR (95% CI) in the other two tertiles. We used three models: unadjusted; adjusted for age, sex, BMI, and Index of Multiple Deprivation; and adjusted for age, sex, BMI, Index of Multiple Deprivation, low mood, anxiety, and multisite chronic pain scores. While we had information on the number of medications used, we did not include this in any of the models, as we perceived it to be a proxy for multimorbidity. We included the low mood, anxiety scores, and the pain measure in a separate model, as it has been hypothesized that these measures could be mediators rather than confounders for the association of sleep disturbance with multimorbidity [30].

For the longitudinal analysis, we investigated the association between wave 1 sleep disturbance in tertiles and the incidence of multimorbidity developed by wave 3 as a count variable (i.e. 0, 1, 2, 3, 4, …, etc.), using multivariable Poisson regression. We were unable to use a time-to-event analysis such as Cox regression because of the limited number of follow-ups (waves 2 and 3). To catch the incidence outcome, we excluded participants who already had two or more long-term conditions at wave 1 as these participants already had multimorbidity. To define the mediators and measure their effects adequately, we used low mood, anxiety, and multisite chronic pain measured at wave 2 rather than wave 1. All other aspects of the longitudinal analysis were the same as the cross-sectional analysis.

For both the cross-sectional and longitudinal analyses, we performed mediation analysis to investigate the relative contribution of both the direct “effect” from sleep disturbance to multimorbidity and the indirect “effect” of sleep disturbance via low mood, anxiety, or multisite chronic pain. Note, that we could only measure how much each variable contributed to the association, this was *not* a measure of causal effects. To do this, we used path analysis, which involves measuring the effect of each hypothesized pathway in a model (in this case, sleep disturbance → low mood, sleep disturbance → anxiety, sleep disturbance → multisite chronic pain, and sleep disturbance, low mood, anxiety, and multisite chronic pain → multimorbidity). With these pathways, we were able to get the results for the direct effect of sleep disturbance on multimorbidity, as well as the indirect effect of sleep disturbance on multimorbidity via low mood, anxiety, and multisite chronic pain. We were able to transform the direct and indirect effects into RR, and we were able to add the effects together to get the total effect, from which we could then estimate the percentage contribution of each effect. All the pathways were adjusted for the same covariates that were used in the standard multivariable Poisson regression analysis. This was done using the generalized Structural Equation Modelling (SEM) function [31], and bootstrap was used to estimate the 95% CIs of the percentage estimates [32].

Most of the analyses were performed using R version 4.1.2., except for the mediation analysis which was performed using STATA 18.

### Sensitivity analyses

To test assumptions regarding our main analysis, we performed some additional sensitivity analyses. Our list of long-term conditions was based on an earlier study of multimorbidity in KPIC^18^, that included sleep problems and psychological problems as health conditions, and we decided to stick as closely as possible to the earlier study for our main analysis. Given that our list of conditions for multimorbidity included sleep problems (exposure in this study) and psychological problems and pain (mediators in this study), there is a risk that any association found between sleep disturbance (adjusting for low mood, anxiety, and multisite chronic pain) and multimorbidity is due to tautology (i.e. morbidities overlapping between exposure and outcome), rather than a real temporal association between exposure (sleep disturbance) and outcome (multimorbidity). Hence, we redid the cross-sectional, longitudinal, and mediation analysis using a definition of multimorbidity that excluded both sleep problems and psychological problems, as well as joint pain, fibromyalgia, chronic fatigue syndrome, and irritable bowel syndrome.

### Patient and public involvement

Three patient and public involvement (PPI) representatives with multimorbidity were involved in this study, particularly during the initial study design process. They provided their inputs, including discussions about the experiences of living with multiple conditions, the lack of research on the temporal relationship between sleep and multimorbidity, and the importance for this study. The protocol for our study was shared with the members in lay-person language for commentary and recommendations.

## Results

### Cross-sectional analysis

At wave 1, 4738 participants returned the questionnaire and 4488 had sufficient sleep data reported. Of the 4488 participants, the mean age was 63.68 years (SD 10.11) and 57 per cent (*n* = 2551) were women.

There were substantial differences between the three sleep disturbance score tertile groups for all measured characteristics (multimorbidity score, age, sex, BMI, medication count, Index of Multiple Deprivation, low mood, and anxiety scores and multisite chronic pain; p-values <.001). Specifically, people in the highest tertile for sleep disturbance tended to have a higher multimorbidity score, be younger, were more likely to be female, were on more medications, and had higher scores for low mood, anxiety, and multisite chronic pain (Table 1).

**Table 1:** Characteristics of participants for cross-sectional analysis at wave 1

	Sleep disturbance			
	Tertile 1	Tertile 2	Tertile 3	P
*N*	1563	1457	1468	
Multimorbidity score, median (IQR)	1 (0–2)	1 (0–2)	1 (1–3)	<.001
Age, years, mean (SD)	64.8 (9.8)	64.0 (10.3)	62.2 (10.1)	<.001
Female sex, % (*n*)	47.4 (733)	57.5 (829)	68.2 (989)	<.001
BMI, kg/m^2^, mean (SD)	26.3 (4.4)	26.9 (4.8)	28.6 (6.3)	<.001
Index of Multiple Deprivation, 1–10, median (IQR)	7 (4–10)	6 (4–10)	5 (3–8)	<.001
Medication % (*n*)				
Any	76.8 (1201)	80.4 (1172)	85.4 (1254)	<.001
> 2	60.8 (951)	66.2 (965)	74.8 (1098)	<.001
> 4	33.2 (519)	42.6 (621)	52.1 (765)	<.001
HADs Anxiety Score, median (IQR)	3 (1–5)	5 (3–7)	8 (5–11)	<.001
HADs Depression Score, median (IQR)^*^	2 (1–3)	3 (2–5)	6 (4–9)	<.001
Multisite chronic pain, range 1–45, median (IQR)	1 (0–4)	3 (0–6)	6 (2–10)	<.001

IQR, interquartile range.

^*^Used to measure low mood.

**Figure 2:**
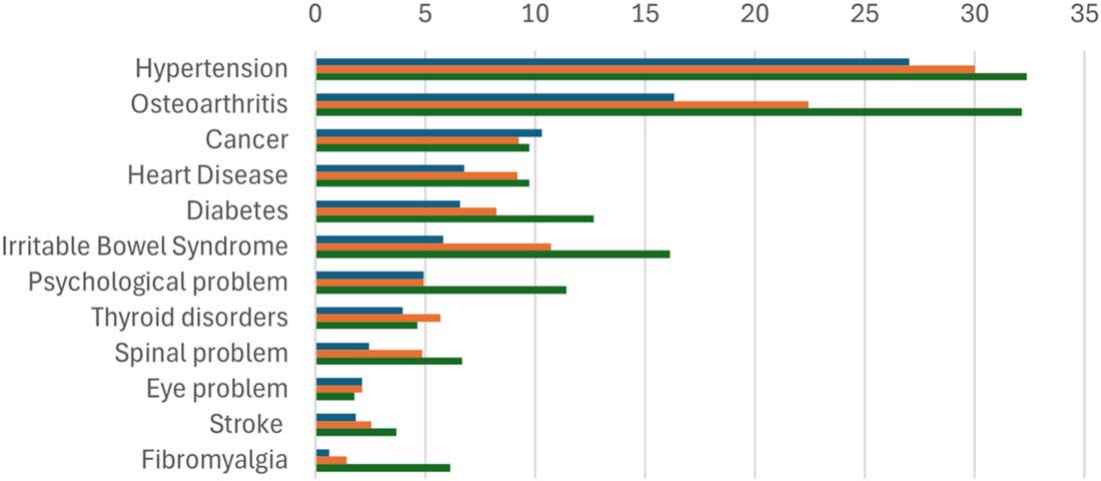
Prevalence (%) of major long-term conditions by sleep disturbance tertile at wave 1. For each of the blocks of columns, the top sub-column (in blue) is the prevalence in the lowest sleep disturbance tertile, the middle sub-column (in orange) is the prevalence in the mid-tertile, and the bottom sub-column (in green) is the prevalence in the highest tertile.

The prevalence of most of the common long-term conditions tended to be higher in higher sleep disturbance tertiles (Fig. 2).

A substantial, dose-dependent association between sleep disturbance and multimorbidity was detected with all three Poisson models (unadjusted, partially adjusted, and fully adjusted). The RR for multimorbidity adjusted for age, sex, BMI, and Index of Multiple Deprivation was 1.23 (95% CI, 1.15–1.32) for T2, and 1.63 (95% CI, 1.52–1.75) for T3 compared with sleep disturbance T1 (reference; p for trend ≤ .001). However, when adding three potential mediators (low mood, anxiety, and multisite chronic pain) into the model, the magnitude of the RRs decreased substantially although the trend remained statistically significant (p for trend ≤ .001 [Table 2]).

**Table 2:** Sleep disturbance and prevalence of multimorbidity at wave 1 (cross-sectional analysis)

Sleep disturbance	Rate ratio (95% confidence interval)		
	Crude	Adjusted^*^	Adjusted^†^
Tertile 1	1 (Ref)	1 (Ref)	1 (Ref)
Tertile 2	1.25 (1.17–1.34)	1.23 (1.15–1.32)	1.09 (1.01–1.18)
Tertile 3	1.67 (1.57–1.78)	1.63 (1.52–1.75)	1.21 (1.11–1.32)
P for trend	<.001	<.001	<.001

^*^Adjusted for age, sex, BMI, and deprivation.

^†^Adjusted for age, sex, BMI, deprivation, anxiety, and low mood scores and multisite chronic pain.

### Longitudinal analysis

Of the 4488 participants at wave 1, 2871 did not have multimorbidity (i.e. two or more conditions) so were at risk for multimorbidity at wave 3, and 1941 of these participants completed the follow-up questionnaires (Fig. 1). The differences in characteristics between the responders and the non-responders can be found in Supplementary Table S1. Of the 1941 participants, 825 were in sleep disturbance T1 at wave 1, 635 were in T2, and 481 were in T3. Again, participants in the highest tertile for sleep disturbance tended to have a higher multimorbidity score, to be younger, were more likely to be female, were on more medications, and had higher scores for low mood and anxiety (Table 3), as in the full wave 1 study group (Table 1).

**Table 3:** Characteristics of participants for longitudinal analysis

	Sleep disturbance at wave 1			
	Tertile 1	Tertile 2	Tertile 3	P
*N*	825	635	481	
Multimorbidity score, median (wave 3, IQR)	1 (0–1)	1 (0–1)	1 (0–1)	<.001
Female sex % (*n*)	49.0 (399)	61.1 (384)	67.2 (320)	<.001
BMI, kg/m^2^, mean (SD)	25.7 (4.0)	26.2 (4.4)	27.2 (5.2)	<.001
Index of Multiple Deprivation, 1–10, median (IQR)	7 (5–10)	6 (4–10)	5 (3–9)	<.001
Medication % (*n*)				
Any	71.5 (590)	78.1 (496)	80.0 (385)	<.001
>2	52.2 (431)	59.4 (377)	64.7 (311)	<.001
>4	21.1 (174)	30.1 (191)	35.6 (171)	<.001
HADs Anxiety Score, median (wave 2;IQR)	3 (1–5)	5 (3–7)	7 (4–10)	<.001
HADs Depression Score, median (wave 2;IQR)^*^	1 (1–3)	3 (1–5)	5 (3–8)	<.001
Multisite chronic pain, range 1–45, median (wave 2;IQR)	1 (0–4)	2 (0–5)	4 (1–9)	<.001

IQR, interquartile range.

^*^Used to measure low mood.

**Figure 3:**
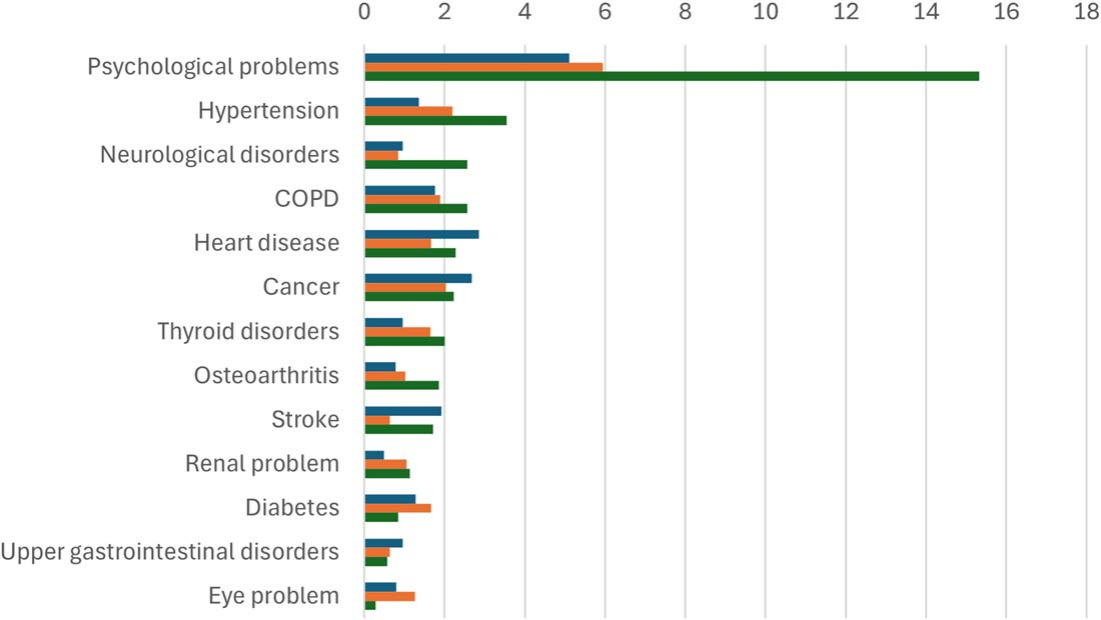
Incidence (%) of major long-term conditions by wave 1 sleep disturbance tertile at wave 3. For each of the blocks of columns, the top sub-column (in blue) is the incidence in the lowest sleep disturbance tertile, the middle sub-column (in orange) is the incidence in the mid-tertile, and the bottom sub-column (in green) is the incidence in the highest tertile.

The incidence of developing common long-term conditions during follow-up tended to be higher in higher sleep disturbance tertiles at wave 1, particularly for psychological problems (Fig. 3).

A substantial, dose-dependent association between sleep disturbance and incident multimorbidity was observed in the unadjusted, partially adjusted and fully adjusted Poisson models. The RRs for the multimorbidity adjusted for age, sex, BMI, and Index of Multiple Deprivation were 1.19 (95% CI; 1.05–1.34) for T2 and 1.40 (95% CI; 1.22–1.60) for T3 compared with sleep disturbance T1 (reference; p for trend ≤ .001). When adding three potential mediators (low mood, anxiety, and multisite chronic pain) into the model, the magnitude of the RRs remained similar (p for trend = .007 [Table 4]).

**Table 4:** Sleep disturbance at wave 1 and incidence of multimorbidity at wave 3 (longitudinal analysis)

Sleep disturbance	Rate ratio (95% confidence interval)		
	Crude	Adjusted^*^	Adjusted^†^
Tertile 1	1 (Ref)	1 (Ref)	1 (Ref)
Tertile 2	1.13 (1.01–1.28)	1.19 (1.05–1.34)	1.12 (0.98–1.28)
Tertile 3	1.32 (1.17–1.49)	1.40 (1.22–1.60)	1.25 (1.06–1.47)
P for trend	<.001	<.001	.007

^*^Adjusted for age, sex, BMI, and deprivation.

^†^Adjusted for age, sex, BMI, deprivation, anxiety, and low mood scores and multisite chronic pain.

### Mediation and interaction analysis

**Figure 4:**
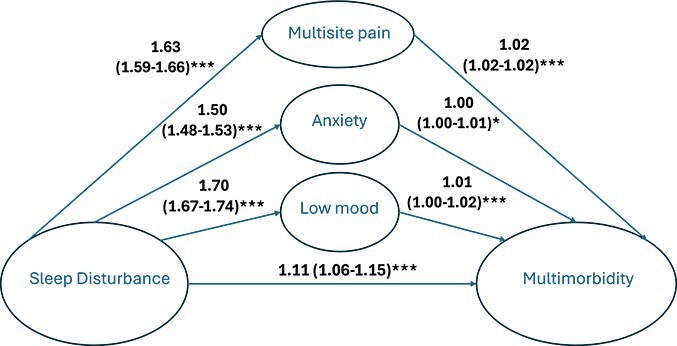
Cross-sectional mediation model showing the relationship between sleep disturbance and multimorbidity, mediated by low mood, anxiety, and multisite chronic pain. This model is showing the mediation results for the prevalence of multimorbidity. All the variables measured were done so at wave 1. The sleep score was categorized into tertiles, multimorbidity was measured as a count variable. All pathways were analyzed using Poisson regression, adjusted for age, sex, BMI, and Index of Multiple Deprivation, and each pathway from mediator to multimorbidity was adjusted for the other two mediators. ^*^p-value >.05, ^**^p-value <.05 and >.001, and ^***^p-value <.001.

**Figure 5:**
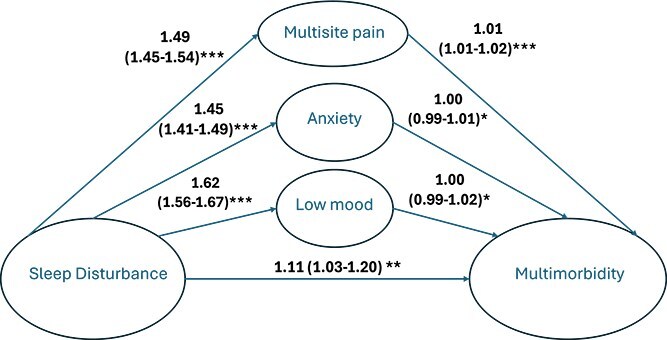
Longitudinal mediation model showing the relationship between sleep disturbance and multimorbidity, mediated by low mood, anxiety, and multisite chronic pain. This model is showing the mediation results for the incidence of multimorbidity. The sleep tertiles were measured at wave 2, the mediators were measured at wave 2 and newly developed multimorbidity since wave 1 was measured and counted until wave 3. All pathways were adjusted for age, sex, BMI, and Index of Multiple Deprivation, and each pathway from mediator to multimorbidity was adjusted for the other two mediators. ^*^p-value >.05, ^**^p-value <.05 and > .001, and ^***^p-value <.001.

For the cross-sectional data (see Fig. 4), 14 per cent (95% CI; 9% to 19%) of the association between sleep disturbance and multimorbidity was indirectly obtained (i.e. mediated) through multisite chronic pain and 7 per cent (95% CI; 2% to 13%) was mediated through low mood. There was no evidence of substantial mediation through anxiety. For the longitudinal analysis (see Fig. 5), 10 per cent (95% CI; 1% to 18%) of the association between sleep and multimorbidity was mediated through multisite chronic pain (Table 5).

**Table 5:** Direct and indirect association (through low mood, anxiety, or pain) between sleep disturbance and multimorbidity

Variables	Cross-sectional (95% CI)	Longitudinal (95% CI)
Sleep disturbance (direct effect %)	77 (66,87)	86 (72100)
Low mood (mediator %)	7 (2,13)	4 (-5,12)
Anxiety (mediator %)	2 (−1,5)	0 (−5,6)
Pain (mediator %)	14 (9,19)	10 (1,18)

CI, confidence intervals.

### Sensitivity analyses

After removing conditions related to sleep problems, low mood, anxiety, and multisite chronic pain from the multimorbidity outcome, the results remained the same in terms of direction and significance of association for both the cross-sectional (Supplementary Table S2) and longitudinal (Supplementary Table S3) analyses. Similarly, when performing mediation analysis without these conditions, similar results were observed for the cross-sectional and longitudinal analyses (Supplementary Table S4).

## Discussion

In this population-based cohort study, we found evidence that sleep disturbance was associated with multimorbidity in a dose-dependent manner in both cross-sectional and longitudinal analyses, even after removing sleep and psychological problems from our multimorbidity score. After adjusting for low mood, anxiety, and pain, the magnitude of the association was reduced in the cross-sectional analysis, but not the longitudinal analysis. In addition, we found that low mood and pain, but not anxiety, significantly mediated 7 per cent and 14 per cent, respectively, of the association between sleep disturbance and concurrent multimorbidity (prevalence), and pain mediated 10 per cent of the association between sleep disturbance and future multimorbidity (incidence).

The results are consistent with the findings from some previous studies on the association between sleep and multimorbidity. Nicholson et al. [33] found that compared to participants with neutral satisfaction regarding their sleep, men who were dissatisfied with their sleep had a higher prevalence of multimorbidity, and women who were satisfied with their sleep had a lower prevalence of multimorbidity, even after adjusting for multiple confounders [33]. Similarly, Guo et al. [34] found that women (but not men) with persistent short sleep duration were more likely to develop multimorbidity after four years of follow-up. Our analysis differs from Guo et al. [34] by including a more comprehensive measure of sleep disturbance that includes more symptoms of sleep disturbance rather than just the length of sleep [19]. For the cross-sectional results, there was a roughly 50 per cent reduction in estimates of the association between sleep disturbance and multimorbidity when low mood, anxiety, and pain were adjusted for, suggesting they are more likely to be mediators in the association, rather than simple confounding factors. Mediation analyses for the cross-sectional association found a partial association between sleep disturbance and multimorbidity indirectly through low mood (7 per cent) and pain (14 per cent). Previously, Smith et al. [30] performed a systematic review and meta-analysis on the association between sleep problems and multimorbidity, where they carried out mediation analysis for the pooled estimates. They found that pain (24 per cent), anxiety (21 per cent), and depression (11.2 per cent) mediated about half of the association between sleep problems and multimorbidity. Similarly, Muhammad et al. [35] found that 4.3 per cent of the association between multimorbidity and sleep problems was mediated by depression symptoms, though pain had a larger mediating role (11.2 per cent). Our results are more consistent with those of Muhammad et al. [35]; however, they did not investigate anxiety as a mediator. Our results are also consistent with the results of a meta-analysis of intervention studies for insomnia in fibromyalgia patients, where benefits are seen for sleep problems, pain, and depression but less consistently for anxiety [16].

The impact of low mood and pain on multimorbidity observed in this study are consistent with the central pain sensitization hypothesis of chronic health conditions [36]. Pain sensitization is known to vary from person to person and has been shown to have a genetic component [37, 38]. It has been hypothesized that individuals with a greater level of pain sensitization are more likely to be diagnosed with chronic health conditions, such as osteoarthritis and fibromyalgia, earlier than those with lower levels of pain sensitization [39]. It has also been hypothesized that depression (and other central pain hypersensitivity traits) and multimorbidity are shared consequences of biological aging (with depression possibly being linked to neuronal degeneration) [9]. Previous research had also suggested that central pain sensitivity could be increased by a lack of delta-sleep [5], a specific pattern of deep non-rapid eye movement sleep believed to be restorative [5]. This has been hypothesized to be due to hyperactivity in the thalamus interfering with the ability of delta-sleep to weaken the synapses linked to pain signals [40]. It has also been hypothesized that sleep disturbance leads to decreases in emotional regulation (leading to increased depressive symptoms) [41], that melatonin (a key sleep inducing hormone) has anti-depressant effects [42] and that sleep facilitates the release of endorphins [43]. Research has also suggested that that the association between sleep disturbance and central pain sensitivity could be genetically mediated [38, 44] and due to inflammatory [42, 43] processes (which could also explain the association with multimorbidity [45]). Our longitudinal analysis has found that multisite chronic pain (a characteristic of central pain sensitization) acts as a mediator in the association between sleep disturbance and multimorbidity.

### Strengths and limitations

Our study was able to investigate both the prevalence and incidence of multimorbidity in a community sample. This allowed us to measure both concurrent and predictive effects of sleep disturbance on multimorbidity. We included low mood and anxiety as separate covariables in our analysis, allowing for the relationship between sleep disturbance and different psychological factors to be investigated with regard to multimorbidity. Our findings remain reasonably consistent after removing the conditions related to sleep disturbance and multisite chronic pain from the multimorbidity score. However, there are limitations to the study. One recurring issue in studies around multimorbidity is in how multimorbidity is defined. A systematic review by Ho et al. [46] found that 36.4 per cent of studies did not give a reference definition of multimorbidity, and 12.9 per cent did not even mention what health conditions were used. This lack of transparency in how multimorbidity is defined can make it difficult for researchers and clinicians to make meaningful conclusions. In this study, we counted multimorbidity from any long-term conditions onwards as our outcome and used Poisson regression, a regression model developed for outcomes in count variables to estimate the relative risk. This has avoided an arbitrary cut-off for multimorbidity and makes the risk estimate more robust. However, this is a relatively small cohort study undertaken in just one region of the UK, with <1000 participants per sleep disturbance tertile in the longitudinal analysis. Future studies of larger cohorts, involving more widespread geographical regions of the UK, would strengthen the reliability and generalizability of the results. A cohort with more follow-up time-points and time-to-event measures also would allow for a more thorough assessment. Another big issue is the dropout of participants during follow-up, with only 51 per cent of the wave 1 participants completing the questionnaire by wave 3, including 67 per cent of participants in the highest sleep tertile. This will have reduced the power as well as the generalizability of the findings for the longitudinal analyses and may have introduced survivor bias into the analyses. With previous cohort studies, it has been found that participants who volunteer for the cohort are often less socially deprived and less likely to be obese, and to smoke and drink less heavily, compared to the general population [47]. This may introduce selection bias into the analyses, making the results less applicable to the general population. We could not completely exclude such bias, even though KPIC is a community cohort of mostly White British people aged over 40 years from the selected General Practitioner (GP) practices irrespective of multimorbidity.

In conclusion, we found that sleep disturbance is associated with both prevalent and incident multimorbidity in a dose-dependent manner, and the association is partially mediated by low mood and pain. Further studies on causal pathways and mediators between sleep and multimorbidity are warranted to identify targets for intervention.

## Supplementary Material

KPIC_Supplementary_Material_Sleep_advances_final_zpaf039

## Data Availability

Data access requests should be directed to WZ in the first instance. The KPIC study was registered on the clinicaltrials.gov portal: NCT02098070. The study analysis plan for this manuscript was not preregistered. We developed novel code using ChatGPT to perform mediation analysis. All code is available on request.
