# Supplementary material for: Sleep disturbance and multimorbidity: a cross-sectional and longitudinal study in the knee pain and related health in the community cohort
Source: Sleep Adv. 2025 Jun 11;6(3):zpaf039. doi: 10.1093/sleepadvances/zpaf039 (PMC12413860; doi:10.1093/sleepadvances/zpaf039)
Supplement: KPIC_Supplementary_Material_Sleep_advances_final_zpaf039 [file kpic_supplementary_material_sleep_advances_final_zpaf039.docx]

**Sleep disturbance and multimorbidity: a cross-sectional and longitudinal study in the Knee Pain and related health in the Community (KPIC) cohort**

Will Thompson^1,2^, Subhashisa Swain^1,2,3^, Carol Coupland^4^, Frances Rees^1,5^, Phil Courtney^5^, Michelle Hall^6^, Eamonn Ferguson^6^, David A Walsh^1,2,7^, Ana M Valdes^1,2,7^, Richard Morriss^8^, Michael Doherty^1,2,7^, Weiya Zhang^1,2,7^

1. Academic Rheumatology, Injury Recovery and Inflammation Sciences, School of Medicine, Clinical Sciences Building, Nottingham City Hospital, Hucknall Road, Nottingham, NG5 1PB, United Kingdom
2. Pain Centre Versus Arthritis, University of Nottingham, Nottingham, UK
3. School of Medicine, David Weatherell Building, Keele University, Staffordshire, ST5 5BG, United Kingdom
4. Centre for Academic Primary Care, School of Medicine, University Park, Nottingham, NG7 2RD, United Kingdom
5. Nottingham University Hospitals Trust, Nottingham, UK
6. School of Psychology, University of Nottingham, NG7 2RD, United Kingdom
7. NIHR Nottingham Biomedical Research Centre, Nottingham, UK
8. Institute for Mental Health, University of Nottingham, Nottingham, UK

*Corresponding author

Correspondence at: Academic Rheumatology, Injury Recovery and Inflammation Sciences, School of Medicine, Clinical Sciences Building, Nottingham City Hospital, Hucknall Road, Nottingham, NG5 1PB, United Kingdom

0115 823 1750

weiya.zhang@nottingham.ac.uk

**Supplementary Table S1: Comparison between responders and non-responders for the longitudinal study**

|  | Responders | Non-responders | P-value |
| --- | --- | --- | --- |
| N | 1941 | 930 |  |
| Age, years, mean (SD) | 62.1 (9.8) | 60.8 (10.7) | 0.001 |
| Female sex, % (n) | 57.5 (1103) | 55.5 (512) | 0.339 |
| BMI, kg/m2, mean (SD) | 26.3 (4.49) | 26.7 (5.1) | 0.017 |
| Index of Multiple Deprivation, 1-10, median (IQR) | 6 (4-10) | 6 (3-9) | <0.001 |
| Medication %, (n) |  |  |  |
| Any | 75.8 (1471) | 70.0 (651) | 0.001 |
| > 2 | 57.7 (1119) | 52.4 (487) | <0.001 |
| > 4 | 27.6 (536) | 25.6 (238) | 0.272 |
| HADs Anxiety Score, median (Wave 1, IQR) | 4 (2-7) | 5 (3-8) | <0.001 |
| HADs Depression Score, median (Wave 1, IQR) | 2 (1-5) | 3 (1-6) | <0.001 |
| Multisite pain, 1-45, median (Wave 1, IQR) | 2 (0-5) | 2 (0-5) | 0.499 |

**Supplementary Table S2: Sleep disturbance and prevalence of multimorbidity** **after removing sleep, low mood, anxiety and multisite pain related conditions from outcome at Wave 1 (cross-sectional analysis)**

| Sleep disturbance | Rate ratio (95%confidence interval)  Crude Adjusted^1^ Adjusted^2^ | | |
| --- | --- | --- | --- |
| Tertile 1 | 1 (Ref) | 1 (Ref) | 1 (Ref) |
| Tertile 2 | 1.21 (1.12-1.30) | 1.19 (1.10-1.28) | 1.09 (1.00-1.18) |
| Tertile 3 | 1.49 (1.39-1.59) | 1.49 (1.38-1.60) | 1.19 (1.09-1.31) |
| P for trend | <0.001 | <0.001 | <0.001 |

1. Adjusted for age, sex, BMI and deprivation.
2. Adjusted for age, sex, BMI, deprivation, anxiety and low mood scores and multisite pain.

**Supplementary Table S3: Sleep disturbance at Wave 1 and incidence of multimorbidity Wave 3 after removing sleep, low mood, anxiety and multisite pain related conditions from the outcome (longitudinal analysis)**

| Sleep disturbance | Rate ratio (95%confidence interval)  Crude Adjusted^1^ Adjusted^2^ | | |
| --- | --- | --- | --- |
| Tertile 1 | 1 (Ref) | 1 (Ref) | 1 (Ref) |
| Tertile 2 | 1.12 (1.00-1.27) | 1.18 (1.04-1.34) | 1.13 (0.99-1.30) |
| Tertile 3 | 1.27 (1.12-1.44) | 1.36 (1.18-1.57) | 1.27 (1.08-1.51) |
| P for trend | <0.001 | <0.001 | 0.005 |

1. Adjusted for age, sex, BMI and deprivation.
2. Adjusted for age, sex, BMI, deprivation, anxiety and low mood scores and multisite pain.

**Supplementary Table S4: Direct and indirect association (through low mood, anxiety or pain) between sleep disturbance and multimorbidity, after removing sleep, low mood, anxiety and multisite pain related conditions from the outcome**

| Variables |  | Cross-sectional  (95% CI) | Longitudinal  (95% CI) |
| --- | --- | --- | --- |
| Sleep disturbance (direct effect %) |  | 79 (68,90) | 90 (79,101) |
| Low mood (mediator %) |  | 8 (2,15) | 1 (-7,10) |
| Anxiety (mediator %) |  | -1 (-5,2) | -0 (-6,6) |
| Pain (mediator %) |  | 14 (8,20) | 9 (1,16) |

CI, Confidence Intervals
